# Independent Predictors of Repeat Emergency Room Presentations: Insights from a Cohort of 1066 Consecutive Patients with Non-Cardiac Chest Pain Generating 4770 Repeat Presentations

**DOI:** 10.3390/jcm12165290

**Published:** 2023-08-14

**Authors:** Aleem Khand, Thomas Brankin-Frisby, Matthew Gornall, James Hatherley, Ray Raj, Michael Campbell, Thomas Salmon, Yi-han Yang, Ruth Grainger

**Affiliations:** 1 Liverpool University Hospitals NHS Foundation Trust, Liverpool L9 7AL, UKthomas.salmon@liverpoolft.nhs.uk (T.S.); yi-han.yang@liverpoolft.nhs.uk (Y.-h.Y.); 2 Liverpool Heart and Chest Hospital, Liverpool L14 3PE, UK; 3 Department of Ageing and Chronic Diseases, University of Liverpool, Liverpool L69 3BX, UK; 4 Clinical Trials Unit, University of Liverpool, Liverpool L69 3BX, UK; 5 North-West Coast Academic Science Network, Cheshire WA4 4AB, UK

**Keywords:** acute chest pain, hospitalisation, epidemiology

## Abstract

Background and importance: Chest pain (CP) is one of the most frequent presentations to the emergency department (ED), a large proportion of which is non-cardiac chest pain (NCCP). Repeat attendances to ED are common and impose considerable burden to overstretched departments. Objective: Our aim was to determine drivers for repeat ED presentations using NCCP as the primary cause of index presentation. Design, setting and participants: This was a retrospective cohort study of 1066 consecutive presentations with NCCP to a major urban hospital ED in North England. Index of Multiple Deprivation (IMD), a postcode-derived validated index of deprivation, was computed. Charlson comorbidity index (CCI) was determined by reference to known comorbidity variables. Repeat presentation to ED to any national hospital was determined by a national linked database (population 53.5 million). Independent predictors of ED representation were computed using logistic regression analysis. Results: Median age was 43 (IQR 28–59), and 50.8% were male. Furthermore, 27.8%, 8.1% and 3.8% suffered from chronic obstructive pulmonary disease (COPD), hypertension and diabetes mellitus, respectively. The most frequent diagnoses, using ICD-10 coding, were non-cardiac chest pain (55.1%), followed by respiratory conditions (14.7%). One-year incidence of adjudicated myocardial infarction, urgent or emergency coronary revascularisation and all-cause death was 0.6%, 2% and 5.3%, respectively. There was a total of 4770 ED repeat presentations 1 year prior to or following index presentation with NCCP in this cohort. Independent (multivariate) predictors for frequent re-presentation (defined as ≥2 representations) were a history of COPD (OR [odds ratio] 2.06, *p* = 0.001), previous MI (OR3.6, *p* = 0.020) and a Charlson comorbidity index ≥1 (OR 1.51, *p* = 0.030). The frequency of previous MI was low as only 3% had sustained a previous MI. Conclusions: This analysis indicates that COPD and complex health care needs (represented by high CCI), but not socio-economic deprivation, should be health policy targets for lessening repeat ED presentations. What is already known on this topic: Repeat presentations with non-ischaemic chest pain are common, placing a considerable burden on emergency departments. What this study adds: COPD and complex health care needs, denoted by Charlson comorbidity index, are implicated as drivers for repeat presentation to accident and emergency department. Socio-economic deprivation was not an independent predictor of re-presentation. How might this study affect research, practice, or policy: Community-based support for COPD and complex health care needs may reduce frequency of ED attendance.

## 1. Introduction

Acute chest pain represents 6% of all attendances to emergency departments (ED) and is the most frequent cause for admission to hospital [1]. Repeat presentations to ED are common largely in the absence of serious acute pathology [2]. This places a huge burden on ED that are seldom equipped to deal with long-term pathology that may not require immediate admission to hospital. Frequent flyer is a term used to describe individuals with repeat presentation to ED [2,3]. However, there has been a paucity of data with respect to characterising repeat presentations to ED with chest pain.

Repeat presentations to the emergency department (ED) are common largely in the absence of serious acute pathology [2]. This places a huge burden on EDs that are seldom equipped to deal with long-term pathology that may not require immediate admission to hospital. Moreover, acute chest pain represents 6% of all attendances to EDs and is the most frequent cause for admission to hospital [1]. However, there has been a paucity of data with respect to characterising repeat presentations to the ED with chest pain [4].

Individuals with repeat presentations to ED are often termed frequent flyers [2,3]. Without a clear understanding of the determinants of repeated ED attendance, we are unable to optimally address the needs of frequent flyers in a safe and cost-effective manner.

We prospectively identified patients with non-ischaemic chest pain to ED but subsequently (retrospectively) and comprehensively characterised them in terms of comorbidities, deprivation using IMD (Index of Multiple Deprivation), i.e., an index based on postcode [5,6], final diagnosis and epidemiological characteristics. We had two main aims: to comprehensively characterise this population and their medium-term cardiovascular risk and secondly to grant insight into the drivers for repeat presentation to ED. We hypothesised that both socio-economic deprivation and high burden of disease contributes to repeat ED presentation. Our aim was to identify potential drivers for repeat ED presentation, using NCCP as an example, in order to target these drivers through health policy.

## 2. Methods

From June 2011 to November 2011, all patients visiting University Hospital Aintree emergency department with a predominant symptom of chest pain were prospectively identified with demographic and epidemiological details entered. Aintree University Hospital is a large hospital providing secondary care cardiology with annual attendances of 80,000 per annum to ED. The non-ischaemic cohort were defined as a primary presentation of chest pain where ED practitioners decided not to take a troponin sample. This meant that there was no clinical suspicion of acute coronary syndrome.

The presence or absence of chronic obstructive airways disease (COPD) was not routinely collected at the time of data entry. This was subsequently determined by tracking back up to 5 years with a nationwide linked database (Hospital Episode Statistics—provided by NHS digital) using ICD-10 codes for COPD. The presence of COPD was defined as an ICD-10 admission code for COPD within these 5 years. We extracted coding data for diagnostic codes for all episodes of care.

This study was conducted in concordance with STARD criteria for diagnostic studies.

The cohort was spilt in two depending on frequency of ED presentation. Using the national linked database, which tracks all admissions by reference to a 11-digit unique National Health Service number, at least two additional presentations to ED (for any reason) either the year before or after the index admission was used to define multiple attendees. These frequent attendees were compared to patients with at most only one other presentation to ED excluding the index presentation. Analyses were undertaken to grant an insight into drivers for repeat ED presentation.

## 3. Determination of Charlson Comorbidity Index and Index of Multiple Deprivation

Index of multiple deprivation, a postcode-derived validated index of deprivation, was computed.

Deprivation is based on the LSOA (lower super output area). These are geographical areas of approx. 1500 people, so the deprivation index represents the deprivation of the area rather than the patient. The Indices of Deprivation provide a set of relative measures of deprivation for small areas across England, based on seven domains of deprivation. The domains were combined using the following weights to produce the overall Index of Multiple Deprivation: Income Deprivation (22.5%), Employment Deprivation (22.5%), Education, Skills and Training Deprivation (13.5%), Health Deprivation and Disability (13.5%), Crime (9.3%), Barriers to Housing and Services (9.3%) and Living Environment Deprivation (9.3%) [5,6].

Charlson comorbidity index (CCI) was determined by referring to known comorbidity variables [7,8].

All inpatient data from 2006 onwards (up to but not including index event) was searched for any of the ICD-10 codes listed in Supplementary Materials, Table S1. If there was a Charlson diagnosis code in any episode, a flag was created for that condition. The Charlson comorbidity index (CCI) was then calculated using the scores (Table S1, Supplementary Materials). Charlson comorbidity index includes a diverse number of codes including codes for mental health.

Of the 1066 patients in the non-cardiac chest pain cohort, 111 did not have any hospital admissions pre-index event. A CCI of 0 has been assumed for these patients.

Univariate and multivariate predictors of repeat presentations, either the year before or after index presentation were determined.

### Adjudication of Subsequent Type 1 Myocardial Infarction and Major Adverse Cardiac Event

Using the linked database, admissions with any ischaemic heart disease or chest pain code were retrieved up to 1 year following index presentation with non-ischaemic chest pain. The list of codes used are detailed in Supplementary Materials, Tables S1 and S2, for revascularisation and ischaemic codes, respectively. The presence or absence of biomarker elevation (according to local laboratory reference range) was determined. In case of biomarker elevation independent and blinded (to index event and risk scores) adjudication was undertaken by two experienced clinicians (with a third involved for divergences in adjudication, who acted as a tiebreaker). The criteria for defining MI was based on the 3rd universal definition for MI [9]. Adjudicators were recommended that 50% rise or fall in a 2nd troponin was confirmatory of MI but 20–50% rise or fall was suggestive particularly in the context of convincing ischaemic symptoms or other corroborating evidence. Agreement between clinicians for the presence or absence of myocardial infarction (repeat presentation with biomarker elevation) was 85%. According to Cohen’s kappa (k = 0.58), inter-rater reliability was at the upper limit of the moderate agreement threshold [10].

For patients who had multiple repeat presentations with chest pain, the first presentation was assessed for biomarker elevation and/or adjudication for type 1 MI. If type 1 MI was confirmed, then subsequent admissions/presentations were censored. If the first readmission was negative for type 1 MI or biomarker evaluation, then subsequent presentations with biomarker positive chest pain was adjudicated until a MACE (Major Adverse Cardiac Event) was defined or presentations were exhausted. It is important to note that nationally not all hospitals utilised high-sensitivity troponins.

For all coronary revascularisations (surgical or percutaneous), a single consultant cardiologist adjudicated (by reference to discharge letters, review of coronary angiograms, biochemistry, biomarkers status) if an attempt at revascularisation (coronary artery bypass surgery or percutaneous coronary artery intervention) did occur and if so whether it fulfilled criteria for unplanned coronary revascularisation (defined as hospital admission with unstable angina/acute coronary syndrome and deemed to require inpatient revascularisation inclusive of primary percutaneous coronary interventions).

## 4. Ethics

This manuscript conforms to the ICMJE Recommendations for the Conduct, Reporting, Editing, and Publication of Scholarly Work in Medical Journals.

The project was registered with the hospital research department and the regional ethics board that granted full consent to undertake this study (North–West England regional ethics board). To allow for complete follow-up, special permission was granted, in the absence of individual consent, via a confidential advisory group (UK government home office appointed) for the recruitment of consecutive CP population and collection of data from any hospital nationwide to facilitate the retrieval of clinical records and blood results for patients with possible acute coronary syndrome (15/CAG/0171) [http://www.hra.nhs.uk accessed on 1 May 2023]. This special permission is necessary in the absence of consent by patients. The lack of consent poses an ethical challenge that can only be overcome by demonstrating a clear public good by the proposed research and a lack of feasibility in obtaining individual patient consent in a cohort or population study.

## 5. Statistics

All analyses were performed in Stata v14, StataCorp LLC, 4905 Lakeway Drive College Station, Texas 77845-4512, USA.

Summary statistics were presented as *n* (%) if categorical and as median (IQR) if continuous. For categorical variables, a Pearson χ^2^ test was used to determine *p*-value, except for cases where there were number of MACE events in any category was ≤5, when Fisher’s Exact test was used. For continuous variables, a *t*-test was performed to determine *p* values. A multivariate logistic regression was used on all variables that were significant at the 10% level (*p* < 0.10) for univariate analysis. Death status was also adjusted for within the model. A backwards elimination was used on these variables against the AIC (Akaike Information criteria), to obtain the best fit. A multivariate logistic regression was used on all variables that were significant at the 10% level (*p* < 0.10) in the univariate analysis, this included COPD, hypertension, previous MI, Charlson comorbidity index and deprivation rank. Death status was also adjusted for within the model. A backwards elimination was used on these variables against the AIC criteria, from that the variables COPD, previous MI and Charlson comorbidity index were left in the model to obtain the best fit. Only complete cases were used for the model; hence, there were 930 complete cases. We defined frequent flyers as two presentations to ED. We used this outcome to determine univariate and multivariate predictors. We undertook a sensitivity analysis to understand if the results were altered when only one ED representation (as opposed to two) were used as the endpoint. We re-ran the analyses (univariate and multivariate analysis) of predictors for ED representation.

## 6. Results

A total of 1066 patients with chest pain were identified prospectively as attending ED between June and November 2011, in whom a troponin was not sampled, and the primary diagnosis was non-cardiac. The median age was 43 (IQR 28–59), and 50.8% were male. Furthermore, 27.8% suffered from COPD, 8.1% had a history of hypertension and 3.8% suffered from diabetes mellitus. Most patients self-presented (54.7%) with a substantial proportion arriving via ambulance (42.5%). See Table 1 for baseline characteristics of this cohort.

## 7. Diagnostic Coding at Index Presentation

ICD-10 coding was grouped in systems to summarise the main pathologies and is illustrated in Table 2. The most frequent coding was related to non-cardiac chest pain, followed by respiratory pathology.

## 8. Cardiovascular Outcomes at 1 Year

**Figure 1:**
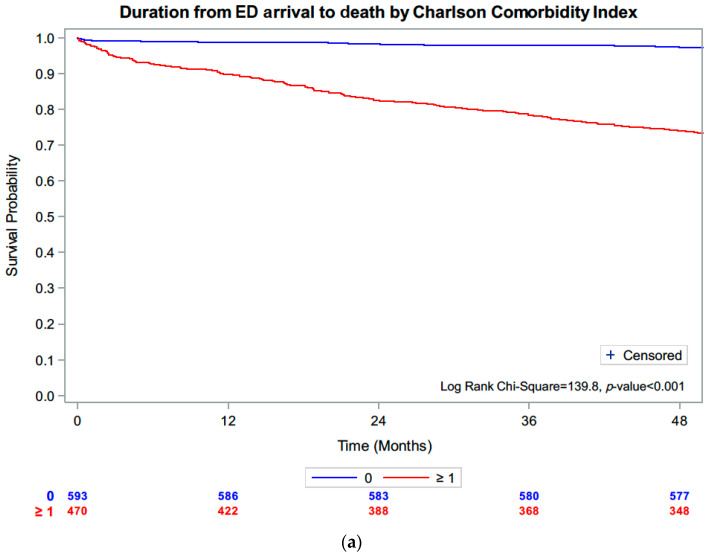

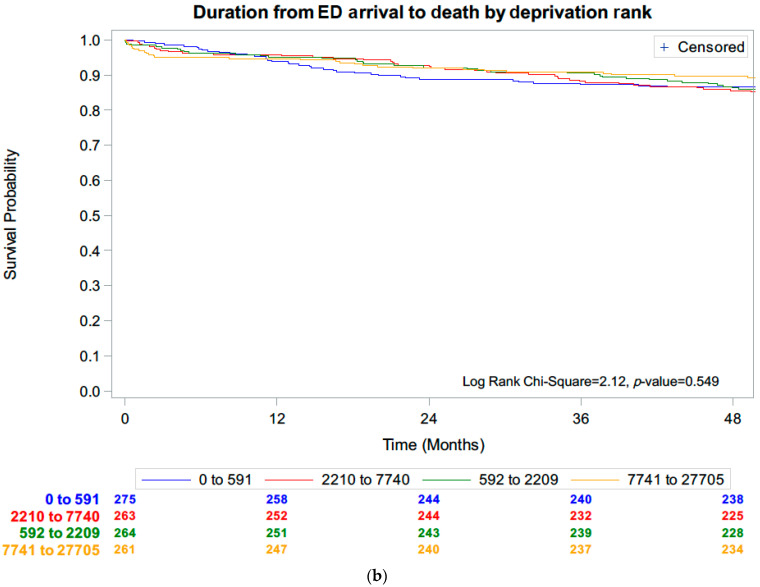
(**a**): Kaplan–Meier survival curve stratified by Charlson comorbidity index. (**b**): Survival according to deprivation rank in quartiles.

One-year incidence of adjudicated myocardial infarction and all-cause death were 0.6% and 5.4%, respectively. In terms of coronary revascularisation, 21 (2.0%) underwent revascularisation, and 0 patients had coronary artery bypass surgery. Therefore, cardiovascular event rates were low, but all-cause death was not insignificant. Death during follow-up was predicted by higher comorbidities (CCI) but not by IMD (Figure 1a,b).

## 9. Frequency of ED Presentation (Table 2)

A total of 1066 patients generated 2754 and 2014 ED presentations in the year before and after index presentation, respectively. The median number of ED presentations were 2.0 (IQR 1.0–5.0). Moreover, 641 (60.3%) had 1–5 ED presentations in the preceding year, and 460 (43.3%) had between 1–5 ED presentations in the year following index presentation. The corresponding proportions for 6–10 additional visits was 6.2% and 3.9% of the cohort in the year before and following index presentation, respectively.

## 10. Comorbidities and Deprivation Index

**Figure 2:**
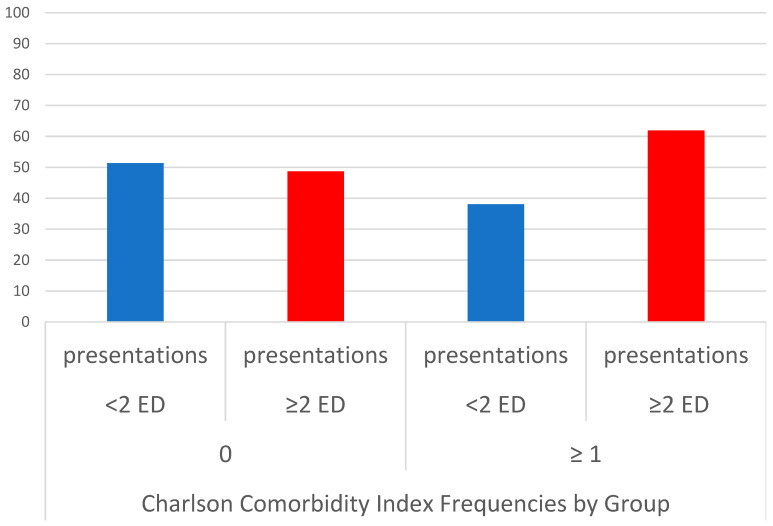
Repeat ED presentations according to Charlson comorbidity index. Abbreviation: ED = emergency department. The *y* axis represents percentage of each subgroup with < or ≥2 repeat ED presentations.

**Figure 3:**
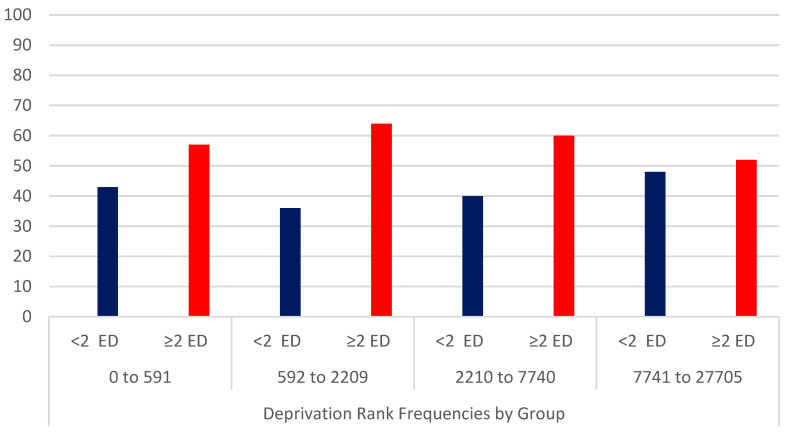
Repeat ED presentations according to deprivation rank. Abbreviation: ED = emergency department. The *y* axis represents percentage of each subgroup with < or ≥2 repeat ED presentations.

For patients with CCI ≥ 1, there were a greater proportion of patients with multiple ED presentation. (Figure 2) The median deprivation index was 2256 (IQR 608-8164). There was no significant difference in the frequency of repeat ED presentations according to deprivation rank (Figure 3).

## 11. Univariate and Multivariate Predictors for Repeat ED Presentations

Table 3 demonstrates univariate associates with repeat ED presentations (≥2 repeat presentations either before or after index NCCP presentation). Significant univariate predictors of repeat ED presentations are emboldened (*p* < 0.05). Table 3 demonstrates the results of multivariate analysis with the best fit for the model. COPD increased the risk of multiple ED visits (≥2 additional to index) by a factor of 2 whereas a CCI of ≥1 increased the risk by 1.5 approximately. A previous MI substantially increased the odds ratio by 3.6 although the number of patients with a previous MI in this cohort was low so the cumulative impact of a history of previous MI would be considerably overshadowed by COPD and the presence of other comorbidities. Sensitivity analysis using only one representation outcome yielded similar results.

COPD, previous MI and CCI continue to retain a significance of *p* < 0.05 as predictors of repeat ED presentation after multivariate analysis, as seen in Table 4. 

## 12. Discussion

This study has several novel findings; deprivation does not have a strong association with repeated ED attendance. Secondly, a CCI score ≥ 1 was linked to both mortality and repeated ED attendance, and thirdly, COPD seems a potent driver for repeat ED attendance. It is also clear that whilst cardiovascular events are low in NCCP cohort, all-cause mortality is still substantial. This analysis suggests repeat attendances are associated with a considerable burden of pathological disease and that resources need to be made available in the community to reduce frequency of ED reattendance. ED attendance for complex health care needs is costly, and furthermore, ED is often inadequate to properly care for these patients.

The strength of our data is the comprehensiveness of follow-up in a national linked database and the use of a postcode-derived index of deprivation to estimate the effects of social disadvantage on ED reattendance. The number of ED reattendances allows a robust exploratory analysis of drivers of repeat ED representation. Community-based support for those with debilitating COPD, for instance, an access to specialist nurses and pharmacists could reduce ED attendance [11].

## 13. Previous Literature

To the authors’ knowledge, no previous studies have examined factors driving repeat ED presentations specifically for patients presenting with NCCP. Although, Mol et al. (2018) did examine representation rate of a cohort in the Netherlands without looking into determining factors [12]. Interestingly, their cohort had a substantially lower ED 1-year representation rate than that of this study (23% and 51%, respectively), although NCCP was determined differently [12]. Perhaps, this large discrepancy indicates superior follow-up of NCCP presenters in the Dutch health care system than in the United Kingdom. Further investigation comparing different management strategies for NCCP presenters with representation rate could therefore yield useful information on the optimum way to treat this patient group.

Looking beyond NCCP, our results have been consistent with previous literature that largely reports a negative association between income (a determinant of deprivation) and repeat ED presentation [4]. Moreover, chronic disease burden is positively associated with increased representation [4].

## 14. Limitations

It was not possible to discern the reason or presenting symptom for repeat ED presentations in our cohort. Understanding the relevance of repeat ED attendance could help further refine strategies for avoidance. We could not adequately explore psychological factors associated with representation to ED as these are not well documented in clinical notes. Also, their accuracy in terms of diagnostic coding is not known. Data on smoking (and other parameters) was incomplete, and we could not discern competing mental health or substance abuse patterns that could complicate the picture. Patients with NCCP are often short-stays, and the system did not force accurate detailed record keeping that could also have helped this research exercise.

The patients were derived from a single centre, even though repeat attendances took place in nearly a dozen ED units. This limits the generalizability of our results and suggests further work is needed to study the external validity of this finding.

Finally, whilst IMD is not an individual assessment of levels of deprivation, it does provide regional information and has been studied extensively [13,14]. Whilst not readily accessible to us, using household income or level of education might have provided a more accurate picture of deprivation. 

## 15. Conclusions

COPD and complex health care needs (represented by high CCI), but not socio-economic deprivation, should be health policy targets for lessening repeat ED representations.

Further prospective cohort studies in multiple centres are warranted, to ensure generalizability of these findings and provide the external validity to this analysis.

## Figures and Tables

**Table 1:** Study baseline characteristics pertinent to risk of acute coronary syndrome.

Age, Median (IQR)	43.0 (28.0–59.0)
Male gender, *n* (%)	541 (50.8%)
Ethnicity (*n*, %), white caucasian	1032 (96.8%)
Non-white	30 (2.8%)
Unknown	4 (0.4%)
Smoking status, *n* (%), current	434 (40.7%)
Never	394 (37.0%)
Ex-smoker < 1 year	30 (2.8%)
Ex-smoker > 1 year	127 (11.9%)
Unknown	78 (7.3%)
COPD, *n* (%)	287 (27.8%)
Hypertension, *n* (%)	84 (8.1%)
Diabetes, *n* (%)	39 (3.8%)
Hyperlipidaemia *n* (%)	23 (2.2%)
History of angina, *n* (%)	38 (3.7%)
History of coronary angioplasty, *n* (%)	1 (0.1%)
Previous MI, *n* (%)	26 (2.5%)
Previous CABG, *n* (%)	7 (0.7%)
Known CAD >50% stenosis on coronary angiography, *n* (%)	4 (0.4%)
Previous CVA/TIA, *n* (%)	13 (1.3%)
Vascular disease, *n* (%)	19 (1.8%)
Valvular heart disease, *n* (%)	5 (0.5%)
Other structural heart disease, *n* (%)	8 (0.8%)
Known heart failure, *n* (%)	6 (0.6%)
Atrial fibrillation/flutter, *n* (%)	13 (1.3%)

**Table 2:** Coded diagnosis, comorbidity, deprivation rank and repeat ED attendances.

Deprivation Rank	
Median (IQR)	2256 (608–8164)
Min, max	5, 27,705
No. available	1032
**Charlson comorbidity index, *n* (%)**	
0	593 (55.8%)
≥1	470 (44.2%)
**Charlson comorbidity index (adjusted by age), *n* (%)**	
0	460 (43.3%)
≥1	603 (56.7%)
**ED visits in the previous year, *n* (%)**	
0	307 (28.9%)
1–5	641 (60.3%)
6–10	66 (6.2%)
11–15	21 (2.0%)
16–20	8 (0.8%)
>20	20 (1.9%)
Min, max	0, 82
**ED visits in the following year, *n* (%)**	
0	524 (49.3%)
1–5	460 (43.3%)
6–10	41 (3.9%)
11–15	14 (1.3%)
16–20	7 (0.7%)
>20	17 (1.6%)
Min, max	0, 43
**Coded diagnosis *n* (%)**	
Non-cardiac chest pain	615 (55.1%)
Respiratory conditions	164 (14.7%)
Endocrine, nervous system, skin, musculoskeletal, GU, unnatural morbidity or mortality	156 (14.0%)
Gastrointestinal	57 (5.1%)
Mental health	29 (2.6%)
Cardiovascular	37 (3.3%)
Cancers, anaemia	2 (0.2%)
Infections	57 (5.1%)

**Table 3:** Univariate correlation with repeat ED presentation.

Variable	Category	Patients	≥2 ED Presentations	Odds Ratio (95% CI)	*p*-Value *
Gender	Male	541	304	Reference category	
	Female	522	314	1.17 (0.91, 1.49)	0.216
Age	NA	1062	618	1.00 (0.99, 1.01)	0.945
COPD	No	776	395	Reference category	
	Yes	287	223	2.66 (1.98, 3.57)	**<0.001**
Diabetes	No	898	509	Reference category	
	Yes	39	28	1.78 (0.90, 3.55)	0.100
Hypertension	No	853	478	Reference category	
	Yes	84	58	1.62 (1.01, 2.60)	**0.043**
History of angina	No	901	512	Reference category	
	Yes	38	26	1.32 (0.69, 2.52)	0.406
Previous MI	No	913	513	Reference category	
	Yes	26	25	4.87 (1.68, 14.11)	**0.004**
High cholesterol	No	917	525	Reference category	
	Yes	23	13	0.88 (0.39, 1.99)	0.761
Valvular heart disease	No	940	539	Reference category	
	Yes	5	3	1.11 (0.19, 6.70)	0.905
Known heart failure	No	939	537	Reference category	
	Yes	6	4	1.50 (0.27, 8.21)	0.642
Charlson comorbidity index	0	593	289	Reference category	
	≥1	470	329	2.45 (1.90, 3.17)	**<0.001**
Deprivation rank	0 to 591	266	151	Reference category	
	592 to 2209	264	169	1.35 (0.96, 1.92)	**0.029**
	2210 to 7740	263	158	1.15 (0.81, 1.62)	0.501
	7741 to 27,705	261	136	0.83 (0.59, 1.17)	**0.020**

* Statistically significant *p*-values at the 5% level are in bold.

**Table 4:** Multivariate analysis for repeat ED presentations.

Variable	Category	Patients	≥2 ED Representations	Odds Ratio (95% CI)	*p*-Value ^
COPD	No	776	395	Reference category	
	Yes	287	223	2.06 (1.34, 3.19)	**0.001**
Previous MI	No	913	513	Reference category	
	Yes	26	25	3.60 (1.22, 10.64)	**0.020**
Charlson comorbidity index	0	593	289	Reference category	
	≥1	470	329	1.51 (1.04, 2.19)	**0.030**

^ Statistically significant *p*-values at the 5% level are in bold.

## Data Availability

To maintain the privacy of our subjects, and under our obligations to the Confidential Advisory Group, further data is not available on request.

## Supplementary Materials

The following supporting information can be downloaded at: https://www.mdpi.com/article/10.3390/jcm12165290/s1, Table S1. ICD 10 chest pains Revascularisation codes used; Table S2. ICD 10 codes used to identify type 1 myocardial infarctions.
