# Supplementary material for: Independent Predictors of Repeat Emergency Room Presentations: Insights from a Cohort of 1066 Consecutive Patients with Non-Cardiac Chest Pain Generating 4770 Repeat Presentations
Source: J Clin Med. 2023 Aug 14;12(16):5290. doi: 10.3390/jcm12165290 (PMC10455527; doi:10.3390/jcm12165290)
Supplement: Supplementary file 1 [file jcm-12-05290-s001.zip › jcm-2457437-supplementary.pdf]

Supplementary tables

Table S1. ICD 10 chest pains Revascularisation codes used

CLINICAL_CODE	CODE_SET_ID	VERSION	SHORT_DESC	LONG_DESC	OPCS_VERSION
K40	OPCS	4	SAPHENOUS VEIN GRAFT REPLACEME	SAPHENOUS VEIN GRAFT REPLACEMENT OF CORONARY ARTERY	4.2
K401	OPCS	4	Saphenous vein graft replaceme	Saphenous vein graft replacement of one coronary artery	4.2
K4010	OPCS	4	Coronary Artery Bypass Grafts	SAPHENOUS VEIN GRAFT REPLACEMENT OF ONE CORONARY ARTERY	4.2
K4010B10	OPCS	4	CORONARY ARTERY BYPASS GRAFTS	SAPHENOUS VEIN GRAFT REPLACEMENT OF ONE CORONARY ARTERY	4.2
K4010B11	OPCS	4	Coronary Artery Bypass Grafts	SAPHENOUS VEIN GRAFT REPLACEMENT OF ONE CORONARY ARTERY	4.2
K4010B14	OPCS	4	CORONARY BYPASS GRAFTS - ENDO	SAPHENOUS VEIN GRAFT REPLACEMENT OF ONE CORONARY ARTERY	4.2
K4010B16	OPCS	4	REPAIR ASC. AORTA & COR. BYPAS	SAPHENOUS VEIN GRAFT REPLACEMENT OF ONE CORONARY ARTERY	4.2
K4010B17	OPCS	4	REPAIR AORTIC ANEURYSM & BYPAS	SAPHENOUS VEIN GRAFT REPLACEMENT OF ONE CORONARY ARTERY	4.2
K4010B18	OPCS	4	CABG & OTHER MAJOR CARDIAC (EG	SAPHENOUS VEIN GRAFT REPLACEMENT OF ONE CORONARY ARTERY	4.2
K4010B1A	OPCS	4	TMR & CABG	SAPHENOUS VEIN GRAFT REPLACEMENT OF ONE CORONARY ARTERY	4.2
K4010B1B	OPCS	4	CABG & CAROTID ENDARTERECTOMY	SAPHENOUS VEIN GRAFT REPLACEMENT OF ONE CORONARY ARTERY	4.2
K4010B1C	OPCS	4	CABG & ABDOMINAL AORTIC ANEURY	SAPHENOUS VEIN GRAFT REPLACEMENT OF ONE CORONARY ARTERY	4.2
K4010B21	OPCS	4	REPLACEMENT/REPAIR OF VALVE(S)	SAPHENOUS VEIN GRAFT REPLACEMENT OF ONE CORONARY ARTERY	4.2
K4010B22	OPCS	4	Coronary Artery Bypass Grafts	SAPHENOUS VEIN GRAFT REPLACEMENT OF ONE CORONARY ARTERY	4.2
K4010B23	OPCS	4	COMPOSITE REPL AORTA,VALVE + C	SAPHENOUS VEIN GRAFT REPLACEMENT OF ONE CORONARY ARTERY	4.2
K402	OPCS	4	Saphenous vein graft replaceme	Saphenous vein graft replacement of two coronary arteries	4.2
K4020	OPCS	4	Coronary Artery Bypass Grafts	SAPHENOUS VEIN GRAFT REPLACEMENT OF TWO CORONARY ARTERIES	4.2
K4020B10	OPCS	4	CORONARY ARTERY BYPASS GRAFTS	SAPHENOUS VEIN GRAFT REPLACEMENT OF TWO CORONARY ARTERIES	4.2
K4020B11	OPCS	4	Coronary Artery Bypass Grafts	SAPHENOUS VEIN GRAFT REPLACEMENT OF TWO CORONARY ARTERIES	4.2
K4020B14	OPCS	4	CORONARY BYPASS GRAFTS - ENDO	SAPHENOUS VEIN GRAFT REPLACEMENT OF TWO CORONARY ARTERIES	4.2
K4020B16	OPCS	4	REPAIR ASC. AORTA & COR. BYPAS	SAPHENOUS VEIN GRAFT REPLACEMENT OF TWO CORONARY ARTERIES	4.2
K4020B17	OPCS	4	REPAIR AORTIC ANEURYSM & BYPAS	SAPHENOUS VEIN GRAFT REPLACEMENT OF TWO CORONARY ARTERIES	4.2
K4020B18	OPCS	4	CABG & OTHER MAJOR CARDIAC (EG	SAPHENOUS VEIN GRAFT REPLACEMENT OF TWO CORONARY ARTERIES	4.2
K4020B1A	OPCS	4	TMR & CABG	SAPHENOUS VEIN GRAFT REPLACEMENT OF TWO CORONARY ARTERIES	4.2
K4020B1B	OPCS	4	CABG & CAROTID ENDARTERECTOMY	SAPHENOUS VEIN GRAFT REPLACEMENT OF TWO CORONARY ARTERIES	4.2
K4020B1C	OPCS	4	CABG & ABDOMINAL AORTIC ANEURY	SAPHENOUS VEIN GRAFT REPLACEMENT OF TWO CORONARY ARTERIES	4.2
K4020B21	OPCS	4	REPLACEMENT/REPAIR OF VALVE(S)	SAPHENOUS VEIN GRAFT REPLACEMENT OF TWO CORONARY ARTERIES	4.2
K4020B22	OPCS	4	CABG + AVR + Carotid Endartere	SAPHENOUS VEIN GRAFT REPLACEMENT OF TWO CORONARY ARTERIES	4.2
K4020B23	OPCS	4	COMPOSITE REPL AORTA,VALVE + C	SAPHENOUS VEIN GRAFT REPLACEMENT OF TWO CORONARY ARTERIES	4.2
K4020B32	OPCS	4	Coronary Artery Bypass Grafts	SAPHENOUS VEIN GRAFT REPLACEMENT OF TWO CORONARY ARTERIES	4.2
K403	OPCS	4	Saphenous vein graft replaceme	Saphenous vein graft replacement of three coronary arteries	4.2
K4030	OPCS	4	Coronary Artery Bypass Grafts	SAPHENOUS VEIN GRAFT REPLACEMENT OF THREE CORONARY ARTERIES	4.2
K4030B10	OPCS	4	CORONARY ARTERY BYPASS GRAFTS	SAPHENOUS VEIN GRAFT REPLACEMENT OF THREE CORONARY ARTERIES	4.2
K4030B11	OPCS	4	Coronary Artery Bypass Grafts	SAPHENOUS VEIN GRAFT REPLACEMENT OF THREE CORONARY ARTERIES	4.2
K4030B14	OPCS	4	CORONARY BYPASS GRAFTS - ENDO	SAPHENOUS VEIN GRAFT REPLACEMENT OF THREE CORONARY ARTERIES	4.2
K4030B16	OPCS	4	REPAIR ASC. AORTA & COR. BYPAS	SAPHENOUS VEIN GRAFT REPLACEMENT OF THREE CORONARY ARTERIES	4.2
K4030B18	OPCS	4	CABG & OTHER MAJOR CARDIAC (EG	SAPHENOUS VEIN GRAFT REPLACEMENT OF THREE CORONARY ARTERIES	4.2

K4030B1A	OPCS	4	TMR & CABG	SAPHENOUS VEIN GRAFT REPLACEMENT OF THREE CORONARY ARTERIES	4.2
K4030B1B	OPCS	4	CABG & CAROTID ENDARTERECTOMY	SAPHENOUS VEIN GRAFT REPLACEMENT OF THREE CORONARY ARTERIES	4.2
K4030B1C	OPCS	4	CABG & ABDOMINAL AORTIC ANEURY	SAPHENOUS VEIN GRAFT REPLACEMENT OF THREE CORONARY ARTERIES	4.2
K4030B21	OPCS	4	REPLACEMENT/REPAIR OF VALVE(S)	SAPHENOUS VEIN GRAFT REPLACEMENT OF THREE CORONARY ARTERIES	4.2
K4030B22	OPCS	4	CABG + AVR + Carotid Endartere	SAPHENOUS VEIN GRAFT REPLACEMENT OF THREE CORONARY ARTERIES	4.2
K4030B23	OPCS	4	COMPOSITE REPL AORTA,VALVE + C	SAPHENOUS VEIN GRAFT REPLACEMENT OF THREE CORONARY ARTERIES	4.2
K4030B42	OPCS	4	Coronary Artery Bypass Grafts	SAPHENOUS VEIN GRAFT REPLACEMENT OF THREE CORONARY ARTERIES	4.2
K404	OPCS	4	Saphenous vein graft replaceme	Saphenous vein graft replacement of four or more coronary ar	4.2
K4040	OPCS	4	Coronary Artery Bypass Grafts	SAPHENOUS VEIN GRAFT REPLACEMENT OF FOUR OR MORE CORONARY AR	4.2
K4040B10	OPCS	4	CORONARY ARTERY BYPASS GRAFTS	SAPHENOUS VEIN GRAFT REPLACE FOUR OR MORE CORONARY ARTERIES	4.2
K4040B11	OPCS	4	CORONARY ARTERY BYPASS GRAFTS	SAPHENOUS VEIN GRAFT REPLACE FOUR OR MORE CORONARY ARTERIES	4.2
K4040B14	OPCS	4	CORONARY BYPASS GRAFTS - ENDO	SAPHENOUS VEIN GRAFT REPLACE FOUR OR MORE CORONARY ARTERIES	4.2
K4040B16	OPCS	4	REPAIR ASC. AORTA & COR. BYPAS	SAPHENOUS VEIN GRAFT REPLACE FOUR OR MORE CORONARY ARTERIES	4.2
K4040B18	OPCS	4	CABG & OTHER MAJOR CARDIAC (EG	SAPHENOUS VEIN GRAFT REPLACE FOUR OR MORE CORONARY ARTERIES	4.2
K4040B1A	OPCS	4	TMR & CABG	SAPHENOUS VEIN GRAFT REPLACE FOUR OR MORE CORONARY ARTERIES	4.2
K4040B1B	OPCS	4	CABG & CAROTID ENDARTERECTOMY	SAPHENOUS VEIN GRAFT REPLACE FOUR OR MORE CORONARY ARTERIES	4.2
K4040B21	OPCS	4	REPLACEMENT/REPAIR OF VALVE(S)	SAPHENOUS VEIN GRAFT REPLACE FOUR OR MORE CORONARY ARTERIES	4.2
K4040B22	OPCS	4	CABG + AVR + CAROTID ENDARTERE	SAPHENOUS VEIN GRAFT REPLACE FOUR OR MORE CORONARY ARTERIES	4.2
K4040B23	OPCS	4	COMPOSITE REPL AORTA,VALVE + C	SAPHENOUS VEIN GRAFT REPLACE FOUR OR MORE CORONARY ARTERIES	4.2
K4040B52	OPCS	4	Coronary Artery Bypass Grafts	SAPHENOUS VEIN GRAFT REPLACEMENT OF FOUR OR MORE CORONARY AR	4.2
K408	OPCS	4	Other specified saphenous vein	Other specified saphenous vein graft replacement of coronary	4.2
K409	OPCS	4	Unspecified saphenous vein gra	Unspecified saphenous vein graft replacement of coronary art	4.2
K41	OPCS	4	OTHER AUTOGRAFT REPLACEMENT OF	OTHER AUTOGRAFT REPLACEMENT OF CORONARY ARTERY	4.2
K411	OPCS	4	Autograft replacement of one c	Autograft replacement of one coronary artery NEC	4.2
K4110	OPCS	4	CABG + AVR + Carotid Endartere	AUTOGRAFT REPLACEMENT OF ONE CORONARY ARTERY NEC	4.2
K4110B10	OPCS	4	CORONARY ARTERY BYPASS GRAFTS	AUTOGRAFT REPLACEMENT OF ONE CORONARY ARTERY NEC	4.2
K4110B11	OPCS	4	Coronary Artery Bypass Grafts	AUTOGRAFT REPLACEMENT OF ONE CORONARY ARTERY NEC	4.2
K4110B1A	OPCS	4	TMR & CABG	AUTOGRAFT REPLACEMENT OF ONE CORONARY ARTERY NEC	4.2
K4110B21	OPCS	4	AUTO REPLACE ONE CON ART	AUTOGRAFT REPLACEMENT OF ONE CORONARY ARTERY NEC	4.2
K4110B22	OPCS	4	CABG + AVR + Carotid Endartere	AUTOGRAFT REPLACEMENT OF ONE CORONARY ARTERY NEC	4.2
K412	OPCS	4	Autograft replacement of two c	Autograft replacement of two coronary arteries NEC	4.2
K4120	OPCS	4	CABG + AVR + Carotid Endartere	AUTOGRAFT REPLACEMENT OF TWO CORONARY ARTERIES NEC	4.2
K4120B10	OPCS	4	CORONARY ARTERY BYPASS GRAFTS	AUTOGRAFT REPLACEMENT OF TWO CORONARY ARTERIES NEC	4.2
K4120B11	OPCS	4	Coronary Artery Bypass Grafts	AUTOGRAFT REPLACEMENT OF TWO CORONARY ARTERIES NEC	4.2
K4120B1A	OPCS	4	TMR & CABG	AUTOGRAFT REPLACEMENT OF TWO CORONARY ARTERIES NEC	4.2
K4120B21	OPCS	4	AUTO REPLACE TWO CON ART	AUTOGRAFT REPLACEMENT OF TWO CORONARY ARTERIES NEC	4.2
K4120B22	OPCS	4	CABG + AVR + Carotid Endartere	AUTOGRAFT REPLACEMENT OF TWO CORONARY ARTERIES NEC	4.2
K413	OPCS	4	Autograft replacement of three	Autograft replacement of three coronary arteries NEC	4.2
K4130	OPCS	4	CABG + AVR + Carotid Endartere	AUTOGRAFT REPLACEMENT OF THREE CORONARY ARTERIES NEC	4.2
K4130B10	OPCS	4	CORONARY ARTERY BYPASS GRAFTS	AUTOGRAFT REPLACEMENT OF THREE CORONARY ARTERIES NEC	4.2
K4130B11	OPCS	4	Coronary Artery Bypass Grafts	AUTOGRAFT REPLACEMENT OF THREE CORONARY ARTERIES NEC	4.2
K4130B1A	OPCS	4	TMR & CABG	AUTOGRAFT REPLACEMENT OF THREE CORONARY ARTERIES NEC	4.2
K4130B21	OPCS	4	AUTO REPLACE THREE CON ART	AUTOGRAFT REPLACEMENT OF THREE CORONARY ARTERIES NEC	4.2
K4130B22	OPCS	4	CABG + AVR + Carotid Endartere	AUTOGRAFT REPLACEMENT OF THREE CORONARY ARTERIES NEC	4.2

K414	OPCS	4	Autograft replacement of four	Autograft replacement of four or more coronary arteries NEC	4.2
K4140	OPCS	4	CABG + AVR + Carotid Endartere	AUTOGRAFT REPLACEMENT OF FOUR OR MORE CORONARY ARTERIES NEC	4.2
K4140B10	OPCS	4	CORONARY ARTERY BYPASS GRAFTS	AUTOGRAFT REPLACEMENT OF FOUR OR MORE CORONARY ARTERIES NEC	4.2
K4140B11	OPCS	4	Coronary Artery Bypass Grafts	AUTOGRAFT REPLACEMENT OF FOUR OR MORE CORONARY ARTERIES NEC	4.2
K4140B1A	OPCS	4	TMR & CABG	AUTOGRAFT REPLACEMENT OF FOUR OR MORE CORONARY ARTERIES NEC	4.2
K4140B21	OPCS	4	AUTO REPLACE FOUR > CON ART	AUTOGRAFT REPLACEMENT OF FOUR OR MORE CORONARY ARTERIES NEC	4.2
K4140B22	OPCS	4	CABG + AVR + Carotid Endartere	AUTOGRAFT REPLACEMENT OF FOUR OR MORE CORONARY ARTERIES NEC	4.2
K418	OPCS	4	Other specified other autograf	Other specified other autograft replacement of coronary arte	4.2
K4180	OPCS	4	TMR & CABG	OTHER SPECIFIED	4.2
K4180B10	OPCS	4	CORONARY ARTERY BYPASS GRAFTS	OTHER AUTOGRAFT REPLACEMENT OF CORONARY ARTERY OS	4.2
K4180B11	OPCS	4	CORONARY ARTERY BYPASS GRAFTS	OTHER AUTOGRAFT REPLACEMENT OF CORONARY ARTERY OS	4.2
K4180B1A	OPCS	4	TMR & CABG	OTHER AUTOGRAFT REPLACEMENT OF CORONARY ARTERY OS	4.2
K419	OPCS	4	Unspecified other autograft re	Unspecified other autograft replacement of coronary artery	4.2
K4190	OPCS	4	TMR & CABG	UNSPECIFIED	4.2
K4190B10	OPCS	4	CORONARY ARTERY BYPASS GRAFTS	OTHER AUTOGRAFT REPLACEMENT OF CORONARY ARTERY UNSPECIFIED	4.2
K4190B11	OPCS	4	CORONARY ARTERY BYPASS GRAFTS	OTHER AUTOGRAFT REPLACEMENT OF CORONARY ARTERY UNSPECIFIED	4.2
K4190B1A	OPCS	4	TMR & CABG	OTHER AUTOGRAFT REPLACEMENT OF CORONARY ARTERY UNSPECIFIED	4.2
K42	OPCS	4	ALLOGRAFT REPLACEMENT OF CORON	ALLOGRAFT REPLACEMENT OF CORONARY ARTERY	4.2
K421	OPCS	4	Allograft replacement of one c	Allograft replacement of one coronary artery	4.2
K422	OPCS	4	Allograft replacement of two c	Allograft replacement of two coronary arteries	4.2
K423	OPCS	4	Allograft replacement of three	Allograft replacement of three coronary arteries	4.2
K424	OPCS	4	Allograft replacement of four	Allograft replacement of four or more coronary arteries	4.2
K428	OPCS	4	Other specified allograft repl	Other specified allograft replacement of coronary artery	4.2
K429	OPCS	4	Unspecified allograft replacem	Unspecified allograft replacement of coronary artery	4.2
K43	OPCS	4	PROSTHETIC REPLACEMENT OF CORO	PROSTHETIC REPLACEMENT OF CORONARY ARTERY	4.2
K431	OPCS	4	Prosthetic replacement of one	Prosthetic replacement of one coronary artery	4.2
K432	OPCS	4	Prosthetic replacement of two	Prosthetic replacement of two coronary arteries	4.2
K433	OPCS	4	Prosthetic replacement of thre	Prosthetic replacement of three coronary arteries	4.2
K434	OPCS	4	Prosthetic replacement of four	Prosthetic replacement of four or more coronary arteries	4.2
K438	OPCS	4	Other specified prosthetic rep	Other specified prosthetic replacement of coronary artery	4.2
K439	OPCS	4	Unspecified prosthetic replace	Unspecified prosthetic replacement of coronary artery	4.2
K44	OPCS	4	OTHER REPLACEMENT OF CORONARY	OTHER REPLACEMENT OF CORONARY ARTERY	4.2
K441	OPCS	4	Replacement of coronary arteri	Replacement of coronary arteries using multiple methods	4.2
K442	OPCS	4	Revision of replacement of cor	Revision of replacement of coronary artery	4.2
K4420	OPCS	4	POST OP ATTENTION TO GRAFTS -	REVISION OF REPLACEMENT OF CORONARY ARTERY	4.2
K4420B15	OPCS	4	POST OP ATTENTION TO GRAFTS -	REVISION OF REPLACEMENT OF CORONARY ARTERY	4.2
K448	OPCS	4	Other specified other replacem	Other specified other replacement of coronary artery	4.2
K449	OPCS	4	Unspecified other replacement	Unspecified other replacement of coronary artery	4.2
K451	OPCS	4	Double anastomosis of mammary	Double anastomosis of mammary arteries to coronary arteries	4.2
K4510B10	OPCS	4	DOUBLE ANASTOMOSIS OF MAMMARY	DOUBLE ANASTOMOSIS OF MAMMARY ARTERIES TO CORONARY ARTERIES	4.2
K4510B11	OPCS	4	DOUBLE ANASTOMOSIS OF MAMMARY	DOUBLE ANASTOMOSIS OF MAMMARY ARTERIES TO CORONARY ARTERIES	4.2
K4510B21	OPCS	4	CABG WITH VALVE REPAIR	DOUBLE ANASTOMOSIS OF MAMMARY ARTERIES TO CORONARY ARTERIES	4.2
K452	OPCS	4	Double anastomosis of thoracic	Double anastomosis of thoracic arteries to coronary arteries	4.2
K453	OPCS	4	Anastomosis of mammary artery	Anastomosis of mammary artery to left anterior descending co	4.2

K4530	OPCS	4	Coronary Artery Bypass Grafts	ANASTOMOSIS OF MAMMARY ARTERY TO LEFT ANTERIOR DESCENDING CO	4.2
K4530B10	OPCS	4	CORONARY ARTERY BYPASS GRAFTS	ANAST MAMMARY ARTERY TO LT ANTERIOR DESCENDING CORON ARTERY	4.2
K4530B11	OPCS	4	CORONARY ARTERY BYPASS GRAFTS	ANAST MAMMARY ARTERY TO LT ANTERIOR DESCENDING CORON ARTERY	4.2
K4530B13	OPCS	4	MIDCAB-MIN INVASIVE BYPASS GRA	ANAST MAMMARY ARTERY TO LT ANTERIOR DESCENDING CORON ARTERY	4.2
K4530B14	OPCS	4	CORONARY BYPASS GRAFTS - ENDO	ANAST MAMMARY ARTERY TO LT ANTERIOR DESCENDING CORON ARTERY	4.2
K4530B16	OPCS	4	REPAIR ASC. AORTA & COR. BYPAS	ANAST MAMMARY ARTERY TO LT ANTERIOR DESCENDING CORON ARTERY	4.2
K4530B18	OPCS	4	CABG & OTHER MAJOR CARDIAC (EG	ANAST MAMMARY ARTERY TO LT ANTERIOR DESCENDING CORON ARTERY	4.2
K4530B1A	OPCS	4	TMR & CABG	ANAST MAMMARY ARTERY TO LT ANTERIOR DESCENDING CORON ARTERY	4.2
K4530B1B	OPCS	4	CABG & CAROTID ENDARTERECTOMY	ANAST MAMMARY ARTERY TO LT ANTERIOR DESCENDING CORON ARTERY	4.2
K4530B21	OPCS	4	REPLACEMENT/REPAIR OF VALVE(S)	ANAST MAMMARY ARTERY TO LT ANTERIOR DESCENDING CORON ARTERY	4.2
K4530B22	OPCS	4	CABG + AVR + CAROTID ENDARTERE	ANAST MAMMARY ARTERY TO LT ANTERIOR DESCENDING CORON ARTERY	4.2
K4530B23	OPCS	4	COMPOSITE REPL AORTA,VALVE + C	ANAST MAMMARY ARTERY TO LT ANTERIOR DESCENDING CORON ARTERY	4.2
K4530B62	OPCS	4	Coronary Artery Bypass Grafts	ANASTOMOSIS OF MAMMARY ARTERY TO LEFT ANTERIOR DESCENDING CO	4.2
K454	OPCS	4	Anastomosis of mammary artery	Anastomosis of mammary artery to coronary artery NEC	4.2
K4540	OPCS	4	Coronary Artery Bypass Grafts	ANASTOMOSIS OF MAMMARY ARTERY TO CORONARY ARTERY NEC	4.2
K4540B10	OPCS	4	CORONARY ARTERY BYPASS GRAFTS	ANASTOMOSIS OF MAMMARY ARTERY TO CORONARY ARTERY NEC	4.2
K4540B11	OPCS	4	Coronary Artery Bypass Grafts	ANASTOMOSIS OF MAMMARY ARTERY TO CORONARY ARTERY NEC	4.2
K4540B12	OPCS	4	Coronary Artery Bypass Grafts	ANASTOMOSIS OF MAMMARY ARTERY TO CORONARY ARTERY NEC	4.2
K4540B14	OPCS	4	CORONARY BYPASS GRAFTS - ENDO	ANASTOMOSIS OF MAMMARY ARTERY TO CORONARY ARTERY NEC	4.2
K4540B16	OPCS	4	REPAIR ASC. AORTA & COR. BYPAS	ANASTOMOSIS OF MAMMARY ARTERY TO CORONARY ARTERY NEC	4.2
K4540B18	OPCS	4	CABG & OTHER MAJOR CARDIAC (EG	ANASTOMOSIS OF MAMMARY ARTERY TO CORONARY ARTERY NEC	4.2
K4540B1A	OPCS	4	TMR & CABG	ANASTOMOSIS OF MAMMARY ARTERY TO CORONARY ARTERY NEC	4.2
K4540B1B	OPCS	4	CABG & CAROTID ENDARTERECTOMY	ANASTOMOSIS OF MAMMARY ARTERY TO CORONARY ARTERY NEC	4.2
K4540B21	OPCS	4	REPLACE/REPAIR VALVE(S) & CABG	ANASTOMOSIS OF MAMMARY ARTERY TO CORONARY ARTERY NEC	4.2
K4540B22	OPCS	4	CABG + AVR + Carotid Endartere	ANASTOMOSIS OF MAMMARY ARTERY TO CORONARY ARTERY NEC	4.2
K4540B23	OPCS	4	COMPOSITE REPL AORTA,VALVE + C	ANASTOMOSIS OF MAMMARY ARTERY TO CORONARY ARTERY NEC	4.2
K455	OPCS	4	Anastomosis of thoracic artery	Anastomosis of thoracic artery to coronary artery NEC	4.2
K456	OPCS	4	Revision of connection of thor	Revision of connection of thoracic artery to coronary artery	4.2
K458	OPCS	4	Other specified connection of	Other specified connection of thoracic artery to coronary ar	4.2
K459	OPCS	4	Unspecified connection of thor	Unspecified connection of thoracic artery to coronary artery	4.2
K46	OPCS	4	OTHER BYPASS OF CORONARY ARTER	OTHER BYPASS OF CORONARY ARTERY	4.2
K463	OPCS	4	Implantation of mammary artery	Implantation of mammary artery into heart NEC	4.2
K468	OPCS	4	Other specified other bypass o	Other specified other bypass of coronary artery	4.2
K469	OPCS	4	Unspecified other bypass of co	Unspecified other bypass of coronary artery	4.2
K49	OPCS	4	TRANSLUMINAL BALLOON ANGIOPLAS	TRANSLUMINAL BALLOON ANGIOPLASTY OF CORONARY ARTERY	4.2
K491	OPCS	4	Percutaneous transluminal ball	Percutaneous transluminal balloon angioplasty of one coronar	4.2
K4910	OPCS	4	LASER ANGIOPLASTY - ONE BALLOO	PERCUTANEOUS TRANSLUMINAL BALLOON ANGIOPLASTY OF ONE CORONAR	4.2
K4910D11	OPCS	4	ABANDONED PTCA	PERCUT TRANSLUMINAL BALLOON ANGIOPLASTY ONE CORONARY ARTERY	4.2
K4910D12	OPCS	4	SIMPLE PTCA - ONE OR TWO BALLO	PERCUT TRANSLUMINAL BALLOON ANGIOPLASTY ONE CORONARY ARTERY	4.2
K4910D21	OPCS	4	SIMPLE PTCA-MORE THAN 2 BALLOO	PERCUT TRANSLUMINAL BALLOON ANGIOPLASTY ONE CORONARY ARTERY	4.2
K4910D34	OPCS	4	PTCA - ONE STENT & ONE BALLOON	PERCUT TRANSLUMINAL BALLOON ANGIOPLASTY ONE CORONARY ARTERY	4.2
K4910D35	OPCS	4	PTCA - 1 STENT, NO BALLOON	PERCUT TRANSLUMINAL BALLOON ANGIOPLASTY ONE CORONARY ARTERY	4.2
K4910D37	OPCS	4	LASER ANGIOPLASTY - ONE BALLOO	PERCUT TRANSLUMINAL BALLOON ANGIOPLASTY ONE CORONARY ARTERY	4.2

K4910D41	OPCS	4	PTCA - 2 OR MORE STENTS	PERCUT TRANSLUMINAL BALLOON ANGIOPLASTY ONE CORONARY ARTERY	4.2
K4910D42	OPCS	4	PTCA - 2 OR MORE STENTS, NO BA	PERCUT TRANSLUMINAL BALLOON ANGIOPLASTY ONE CORONARY ARTERY	4.2
K4910D44	OPCS	4	PTCA WITH STENT & ROTABLATOR	PERCUT TRANSLUMINAL BALLOON ANGIOPLASTY ONE CORONARY ARTERY	4.2
K4910D45	OPCS	4	PTCA WITH STENT - 2+ BALLOONS	PERCUT TRANSLUMINAL BALLOON ANGIOPLASTY ONE CORONARY ARTERY	4.2
K4910D49	OPCS	4	PTCA WITH 2+STENTS (2+ BALLOON	PERCUT TRANSLUMINAL BALLOON ANGIOPLASTY ONE CORONARY ARTERY	4.2
K4910D4B	OPCS	4	LASER ANGIOPLASTY, MORE THAN O	PERCUT TRANSLUMINAL BALLOON ANGIOPLASTY ONE CORONARY ARTERY	4.2
K4910D4C	OPCS	4	LASER ANGIOPLASTY WITH STENT(S	PERCUT TRANSLUMINAL BALLOON ANGIOPLASTY ONE CORONARY ARTERY	4.2
K492	OPCS	4	Percutaneous transluminal ball	Percutaneous transluminal balloon angioplasty of multiple co	4.2
K4920	OPCS	4	PTCA with Stent & Rotablator	PERCUTANEOUS TRANSLUMINAL BALLOON ANGIOPLASTY OF MULTIPLE CO	4.2
K4920D11	OPCS	4	ABANDONED PTCA	PERCUT TRANSLUM BALLOON ANGIOPLASTY MULITPLE CORON ARTERIES	4.2
K4920D12	OPCS	4	SIMPLE PTCA - ONE OR TWO BALLO	PERCUT TRANSLUM BALLOON ANGIOPLASTY MULITPLE CORON ARTERIES	4.2
K4920D21	OPCS	4	SIMPLE PTCA-MORE THAN 2 BALLOO	PERCUT TRANSLUM BALLOON ANGIOPLASTY MULITPLE CORON ARTERIES	4.2
K4920D34	OPCS	4	PTCA - ONE STENT & ONE BALLOON	PERCUT TRANSLUM BALLOON ANGIOPLASTY MULITPLE CORON ARTERIES	4.2
K4920D35	OPCS	4	PTCA - 1 STENT, NO BALLOON	PERCUT TRANSLUM BALLOON ANGIOPLASTY MULITPLE CORON ARTERIES	4.2
K4920D37	OPCS	4	LASER ANGIOPLASTY - 1 BALLOON	PERCUT TRANSLUM BALLOON ANGIOPLASTY MULITPLE CORON ARTERIES	4.2
K4920D41	OPCS	4	PTCA - 2 OR MORE STENTS	PERCUT TRANSLUM BALLOON ANGIOPLASTY MULITPLE CORON ARTERIES	4.2
K4920D42	OPCS	4	PTCA - 2 STENTS, NO BALLOON	PERCUT TRANSLUM BALLOON ANGIOPLASTY MULITPLE CORON ARTERIES	4.2
K4920D44	OPCS	4	PTCA WITH STENT & ROTABLATOR	PERCUT TRANSLUM BALLOON ANGIOPLASTY MULITPLE CORON ARTERIES	4.2
K4920D45	OPCS	4	PTCA WITH STENT - 2+ BALLOONS	PERCUT TRANSLUM BALLOON ANGIOPLASTY MULITPLE CORON ARTERIES	4.2
K4920D49	OPCS	4	PTCA WITH 2+STENTS (2+ BALLOON	PERCUT TRANSLUM BALLOON ANGIOPLASTY MULITPLE CORON ARTERIES	4.2
K4920D4B	OPCS	4	LASER ANGIOPLASTY - 1 BALLOON	PERCUT TRANSLUM BALLOON ANGIOPLASTY MULITPLE CORON ARTERIES	4.2
K4920D4C	OPCS	4	LASER ANGIOPLASTY WITH STENT(S	PERCUT TRANSLUM BALLOON ANGIOPLASTY MULITPLE CORON ARTERIES	4.2
K493	OPCS	4	Percutaneous transluminal ball	Percutaneous transluminal balloon angioplasty of bypass graf	4.2
K4930	OPCS	4	PTCA with Stent & Rotablator	PERCUTANEOUS TRANSLUMINAL BALLOON ANGIOPLASTY OF BYPASS GRAF	4.2
K4930D12	OPCS	4	SIMPLE PTCA - ONE OR TWO BALLO	PERCUT TRANSLUM BALLOON ANGIOPLASTY BYPASS GRAFT CORON ART	4.2
K4930D21	OPCS	4	SIMPLE PTCA-MORE THAN 2 BALLOO	PERCUT TRANSLUM BALLOON ANGIOPLASTY BYPASS GRAFT CORON ART	4.2
K4930D34	OPCS	4	PTCA - ONE STENT & ONE BALLOON	PERCUT TRANSLUM BALLOON ANGIOPLASTY BYPASS GRAFT CORON ART	4.2
K4930D35	OPCS	4	PTCA - 1 STENT, NO BALLOON	PERCUT TRANSLUM BALLOON ANGIOPLASTY BYPASS GRAFT CORON ART	4.2
K4930D41	OPCS	4	PTCA - 2 OR MORE STENTS	PERCUT TRANSLUM BALLOON ANGIOPLASTY BYPASS GRAFT CORON ART	4.2
K4930D44	OPCS	4	PTCA WITH STENT & ROTABLATOR	PERCUT TRANSLUM BALLOON ANGIOPLASTY BYPASS GRAFT CORON ART	4.2
K4930D45	OPCS	4	PTCA WITH STENT - 2+ BALLOONS	PERCUT TRANSLUM BALLOON ANGIOPLASTY BYPASS GRAFT CORON ART	4.2
K4930D49	OPCS	4	PTCA WITH 2+STENTS (2+ BALLOON	PERCUT TRANSLUM BALLOON ANGIOPLASTY BYPASS GRAFT CORON ART	4.2
K494	OPCS	4	Percutaneous transluminal cutt	Percutaneous transluminal cutting balloon angioplasty of cor	4.2
K498	OPCS	4	Other specified transluminal b	Other specified transluminal balloon angioplasty of coronary	4.2
K4980	OPCS	4	PTCA with Stent & Rotablator	OTHER SPECIFIED	4.2
K4980D34	OPCS	4	PTCA - ONE STENT & ONE BALLOON	TRANSLUMINAL BALLOON ANGIOPLASTY OF CORONARY ARTERY OS	4.2
K4980D41	OPCS	4	PTCA - 2 OR MORE STENTS	TRANSLUMINAL BALLOON ANGIOPLASTY OF CORONARY ARTERY OS	4.2
K4980D44	OPCS	4	PTCA WITH STENT & ROTABLATOR	TRANSLUMINAL BALLOON ANGIOPLASTY OF CORONARY ARTERY OS	4.2
K4980D45	OPCS	4	PTCA WITH STENT - 2+ BALLOONS	TRANSLUMINAL BALLOON ANGIOPLASTY OF CORONARY ARTERY OS	4.2
K4980D49	OPCS	4	PTCA WITH 2+STENTS (2+ BALLOON	TRANSLUMINAL BALLOON ANGIOPLASTY OF CORONARY ARTERY OS	4.2
K499	OPCS	4	Unspecified transluminal ballo	Unspecified transluminal balloon angioplasty of coronary art	4.2
K50	OPCS	4	OTHER THERAPEUTIC TRANSLUMINAL	OTHER THERAPEUTIC TRANSLUMINAL OPERATIONS ON CORONARY ARTERY	4.2
K501	OPCS	4	Percutaneous transluminal lase	Percutaneous transluminal laser coronary angioplasty	4.2

K5010	OPCS	4	LASER ANGIOPLASTY - NO BALLOON	PERCUTANEOUS TRANSLUMINAL LASER CORONARY ANGIOPLASTY	4.2
K5010D36	OPCS	4	LASER ANGIOPLASTY - NO BALLOON	PERCUTANEOUS TRANSLUMINAL LASER CORONARY ANGIOPLASTY	4.2
K502	OPCS	4	Percutaneous transluminal coro	Percutaneous transluminal coronary thrombolysis using strept	4.2
K503	OPCS	4	Percutaneous transluminal inje	Percutaneous transluminal injection of therapeutic substance	4.2
K504	OPCS	4	Percutaneous transluminal athe	Percutaneous transluminal atherectomy of coronary artery	4.2
K508	OPCS	4	Other specified other therapeu	Other specified other therapeutic transluminal operations on	4.2
K5080	OPCS	4	PTCA with Rota -1 balloon,1 or	PTDA	4.2
K5080D31	OPCS	4	PTCA WITH ROTA -1 BALLOON,1 OR	OTHER THERAP TRANSLUMINAL OPERATIONS ON CORONARY ARTERY OS	4.2
K5080D32	OPCS	4	CORONARY TEC - ONE BALLOON	OTHER THERAP TRANSLUMINAL OPERATIONS ON CORONARY ARTERY OS	4.2
K5080D33	OPCS	4	DCA - ONE BALLOON	OTHER THERAP TRANSLUMINAL OPERATIONS ON CORONARY ARTERY OS	4.2
K5080D43	OPCS	4	PTCA-2 OR MORE ROTABLATORS	OTHER THERAP TRANSLUMINAL OPERATIONS ON CORONARY ARTERY OS	4.2
K5080D44	OPCS	4	PTCA WITH STENT & ROTABLATOR	OTHER THERAP TRANSLUMINAL OPERATIONS ON CORONARY ARTERY OS	4.2
K5080D46	OPCS	4	PTCA WITH ROTABLATOR - 2+ BALL	OTHER THERAP TRANSLUMINAL OPERATIONS ON CORONARY ARTERY OS	4.2
K5080D47	OPCS	4	CORONARY TEC - 2+ BALLOONS	OTHER THERAP TRANSLUMINAL OPERATIONS ON CORONARY ARTERY OS	4.2
K5080D48	OPCS	4	DCA - 2+BALLOONS	OTHER THERAP TRANSLUMINAL OPERATIONS ON CORONARY ARTERY OS	4.2
K5080D4A	OPCS	4	CORONARY TEC WITH STENT(S) INC	OTHER THERAP TRANSLUMINAL OPERATIONS ON CORONARY ARTERY OS	4.2
K509	OPCS	4	Unspecified other therapeutic	Unspecified other therapeutic transluminal operations on coronary artery	4.2
K75	OPCS	4	PERCUTANEOUS TRANSLUMINAL BALL	PERCUTANEOUS TRANSLUMINAL BALLOON ANGIOPLASTY AND STENTING O	4.2
K751	OPCS	4	Percutaneous transluminal ball	Percutaneous transluminal balloon angioplasty and insertion	4.2
K752	OPCS	4	Percutaneous transluminal ball	Percutaneous transluminal balloon angioplasty and insertion	4.2
K753	OPCS	4	Percutaneous transluminal ball	Percutaneous transluminal balloon angioplasty and insertion	4.2
K754	OPCS	4	Percutaneous transluminal ball	Percutaneous transluminal balloon angioplasty and insertion	4.2
K758	OPCS	4	Other specified percutaneous t	Other specified percutaneous transluminal balloon angioplasty	4.2
K759	OPCS	4	Unspecified percutaneous trans	Unspecified percutaneous transluminal balloon angioplasty an	4.2

Table S2. ICD 10 codes used to identify type 1 myocardial infarctions

CLINICAL_CODE	CODE_SET_ID	VERSION	SHORT_DESC	LONG_DESC
I20	ICD	10	Angina pectoris	Angina pectoris
I200	ICD	10		UNSTABLE ANGINA
I201	ICD	10		ANGINA PECTORIS WITH DOCUMENTED SPASM
I208	ICD	10		OTHER FORMS OF ANGINA PECTORIS
I209	ICD	10		ANGINA PECTORIS, UNSPECIFIED
I21	ICD	10	Acute myocardial infarction	Acute myocardial infarction
I210	ICD	10		ACUTE TRANSMURAL MYOCARDIAL INFARCTION OF ANTERIOR WALL
I211	ICD	10		ACUTE TRANSMURAL MYOCARDIAL INFARCTION OF INFERIOR WALL
I212	ICD	10		ACUTE TRANSMURAL MYOCARDIAL INFARCTION OF OTHER SITES
I213	ICD	10		ACUTE TRANSMURAL MYOCARDIAL INFARCTION OF UNSPECIFIED SITE
I214	ICD	10		ACUTE SUBENDOCARDIAL MYOCARDIAL INFARCTION
I219	ICD	10		ACUTE MYOCARDIAL INFARCTION, UNSPECIFIED
I22	ICD	10	Subsequent myocardial infarction	Subsequent myocardial infarction
I220	ICD	10		SUBSEQUENT MYOCARDIAL INFARCTION OF ANTERIOR WALL
I221	ICD	10		SUBSEQUENT MYOCARDIAL INFARCTION OF INFERIOR WALL

I228	ICD	10		SUBSEQUENT MYOCARDIAL INFARCTION OF OTHER SITES
I229	ICD	10		SUBSEQUENT MYOCARDIAL INFARCTION OF UNSPECIFIED SITE
I24	ICD	10	Other acute ischaemic heart disease	Other acute ischaemic heart disease
I240	ICD	10		CORONARY THROMBOSIS NOT RESULTING IN MYOCARDIAL INFARCTION
I241	ICD	10		DRESSLER'S SYNDROME
I248	ICD	10		OTHER FORMS OF ACUTE ISCHAEMIC HEART DISEASE
I249	ICD	10		ACUTE ISCHAEMIC HEART DISEASE, UNSPECIFIED
I25	ICD	10	Chronic ischaemic heart disease	Chronic ischaemic heart disease
I250	ICD	10		ATHEROSCLEROTIC CARDIOVASCULAR DISEASE, SO DESCRIBED
I251	ICD	10		ATHEROSCLEROTIC HEART DISEASE
I253	ICD	10		ANEURYSM OF HEART
I254	ICD	10		CORONARY ARTERY ANEURYSM
I255	ICD	10		ISCHAEMIC CARDIOMYOPATHY
I256	ICD	10		SILENT MYOCARDIAL ISCHAEMIA
I46	ICD	10	Cardiac arrest	Cardiac arrest
I460	ICD	10		CARDIAC ARREST WITH SUCCESSFUL RESUSCITATION
I461	ICD	10		SUDDEN CARDIAC DEATH, SO DESCRIBED
I469	ICD	10		CARDIAC ARREST, UNSPECIFIED
R071	ICD	10		CHEST PAIN ON BREATHING
R072	ICD	10		PRECORDIAL PAIN
R073	ICD	10		OTHER CHEST PAIN
R074	ICD	10		CHEST PAIN, UNSPECIFIED
R96	ICD	10	Other sudden death, cause unkn	Other sudden death, cause unkn
R960	ICD	10		INSTANTANEOUS DEATH
R961	ICD	10		DEATH OCCURRING LESS THAN 24 HOURS FROM ONSET OF SYMPTOMS, N
R98X	ICD	10		UNATTENDED DEATH
R99X	ICD	10		OTHER ILL-DEFINED AND UNSPECIFIED CAUSES OF MORTALITY
